# Singular Case of Twins

**Published:** 1848-04

**Authors:** 


*Singular Case of Twins.*—Mr. Hill presented a blighted fœtus, apparently about the fifth month, which was born thirty-six hours before a fully-developed healthy fœtus. He gave the following report of the case:—

I was requested to see Mrs. J., a middle-aged woman, the mother of several children, the gentleman in attendance thinking it was the shoulder that presented; but upon a more careful examination, (the labour having proceeded somewhat,) we discovered that it was the head of a child, apparently about four months. The membranes were ruptured, and the child expelled. The labour pains then subsided, and it was about thirty-six hours after that a second child of the ordinary size, and alive, was born. The placenta was double, and did not differ from that of an ordinary twin case.

*Prov. Med. and Surg. Journ.*

